# An Unusual Presentation of Urinary Retention in a Young Male Patient With Neurofibromatosis Type 1

**DOI:** 10.7759/cureus.73319

**Published:** 2024-11-09

**Authors:** Omar Desouky, Baraa Chkir, Thihnaan Zuhair

**Affiliations:** 1 Urology, Royal Preston Hospital, Lancashire Teaching Hospitals National Health Service (NHS) Foundation Trust, Preston, GBR; 2 Urology, Furness General Hospital, Barrow-in-Furness, GBR; 3 Internal Medicine, North Manchester General Hospital, Manchester University National Health Service (NHS) Foundation Trust, Manchester, GBR

**Keywords:** acute urinary retention (aur), neurofibromatosis type 1, pelvic mass, prostate, sarcoma, urinary retention (ur)

## Abstract

Acute urinary retention (AUR) in young adults is an uncommon presentation often signalling an underlying pathology, particularly when associated with genetic conditions like neurofibromatosis type 1 (NF1). We report the case of a 25-year-old male with a known family history of NF1, who presented with acute urinary retention and progressive pelvic pain. Physical examination was notable for axillary freckling and café-au-lait spots, consistent with NF1. Imaging revealed a large heterogeneous pelvic mass displacing the bladder and rectum. This case emphasises the importance of considering rare aetiologies for acute urinary retention in young males.

## Introduction

Acute urinary retention (AUR) in young adults is relatively uncommon and typically signals an underlying pathology such as anatomic obstruction, neurological dysfunction, or inflammatory conditions [1]. While AUR frequently occurs in older men due to benign prostatic hyperplasia, cases in younger individuals warrant careful evaluation for other aetiologies, particularly in the presence of systemic symptoms or a notable family history of genetic disorders [1].

Pelvic masses causing urinary retention are rare but are documented in cases involving genitourinary, gastrointestinal, or neurogenic tumours [1]. Neurofibromatosis type 1 (NF1), is a common autosomal dominant genetic disorder characterised by mutations in the NF1 gene on chromosome 17 [2]. NF1 affects, approximately, between one in 3,000 and one in 6,000 individuals worldwide and manifests with a wide spectrum of clinical features, including café-au-lait spots, axillary or inguinal freckling, Lisch nodules, and neurofibromas [3].

A significant complication of NF1 is the predisposition to tumour development, both benign and malignant [4]. Malignant peripheral nerve sheath tumours (MPNSTs) are one of the most severe malignancies associated with NF1. These aggressive sarcomas often arise from pre-existing plexiform neurofibromas and carry a poor prognosis due to their high recurrence rate and potential for metastasis [5]. Symptoms of neurogenic or compressive urinary retention in NF1 patients may often overlap with more common causes, complicating the diagnostic pathway [6]. Literature on pelvic masses presenting as AUR in NF1 is sparse, with limited case reports describing how these lesions might mimic more benign causes of obstruction [6].

This case highlights a unique presentation in which the patient’s symptoms of progressive pelvic pain, nocturia, and constipation, culminating in AUR, were ultimately attributable to a large pelvic mass. Given the patient’s family history of NF1, the differential diagnosis initially included neurogenic tumours, which informed the diagnostic approach and eventual management.

## Case presentation

A 25-year-old male patient presented to the emergency department with acute urinary retention. He reported a three to four month history of progressive pelvic pain managed with co-codamol. Additional symptoms included tenesmus, intermittent constipation treated with laxatives, nocturia, and difficulty voiding, culminating in urinary retention. There was no history of hypoaesthesia, paraesthesias, numbness, gait disturbances, haematuria, weight loss, or other neurological symptoms.

The patient had a significant family history of NF1 and was awaiting a neurology evaluation. He was an occasional smoker with no prior surgeries, regular medications, or known allergies.

On physical examination, he exhibited axillary freckling and café-au-lait spots consistent with NF1 but no cutaneous neurofibromas were identified. Abdominal examination was unremarkable. Genital examination revealed normal male external genitalia with bilateral descended testes and an uncircumcised penis. A digital rectal examination revealed a hard, non-mobile pelvic mass anterior to the rectum, with mucosa not fixed to the underlying mass. He was seen by the neurology team and a diagnosis of NF1 was confirmed. 

Serum prostate-specific antigen (PSA) and testicular tumour markers were normal at the time of admission.

**Figure 1:**
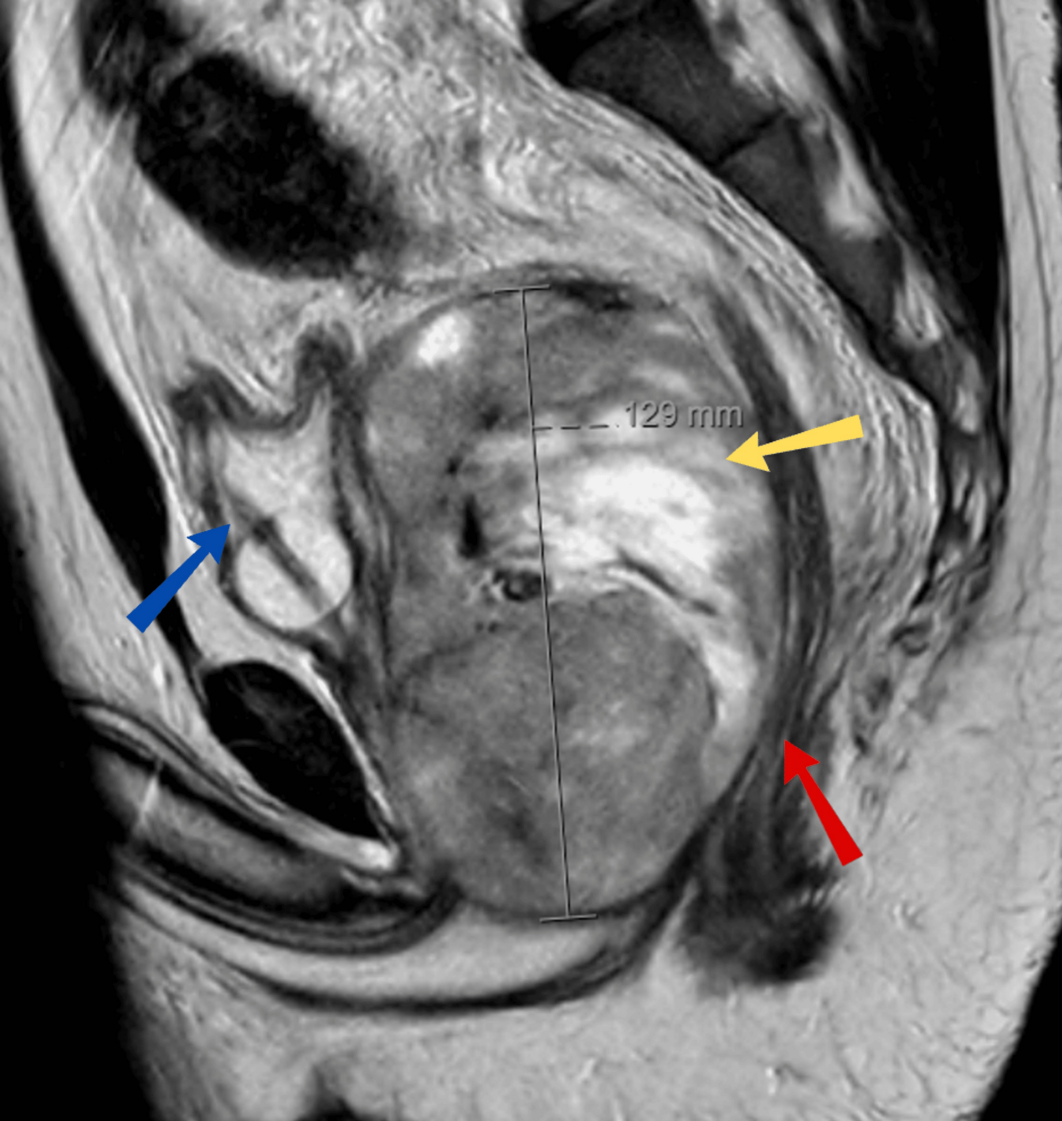
MRI pelvis with contrast demonstrating a large mass (yellow arrow) between the bladder (blue arrow) and rectum (red arrow)

Imaging studies were conducted to evaluate the symptoms. A pelvic magnetic resonance imaging (MRI) scan revealed a 13 cm × 9 cm heterogeneous mass replacing the prostate, effacing the left seminal vesicle, and stretching the rectum over the mass (Figure 1). The mass appeared to displace the bladder anteriorly and the rectum posteriorly without obvious invasion. A staging computed tomography (CT) scan of the chest, abdomen, and pelvis showed no evidence of distant metastasis.

The case was discussed at a multidisciplinary team meeting, and the patient was referred to a specialised sarcoma centre for further management. He underwent total pelvic exenteration with ileal conduit and colostomy formation.

## Discussion

Urinary retention in young males is an uncommon clinical scenario and can often signal underlying pathology that warrants thorough investigation [1]. Common causes in this demographic include urethral strictures, neurological disorders, and infections, however, the presence of a pelvic mass causing compression of the urinary tract is a rare but significant cause that should not be overlooked [1].

NF1 is a genetic disorder characterised by the development of multiple neurofibromas throughout the body [7]. While cutaneous manifestations are most common, internal plexiform neurofibromas can occur and may lead to the formation of large pelvic masses [7-9]. These masses can exert pressure on adjacent organs, leading to symptoms such as pelvic pain, constipation, and urinary retention [8,9].

In our case, the patient presented with acute urinary retention following months of pelvic discomfort and urinary difficulties. The absence of common causes of urinary retention in young males prompted further imaging studies, which revealed a large pelvic mass replacing the prostate. This highlights the importance of considering pelvic masses as a potential cause of urinary retention, especially in patients with underlying conditions like NF1.

Pelvic neurofibromas in NF1 patients can vary in presentation. They may be asymptomatic or cause symptoms due to mass effect [7]. Compression of the bladder neck or urethra by a pelvic mass can lead to obstructive urinary symptoms, these masses may infiltrate surrounding structures, complicating surgical management [7,9,10].

Imaging modalities such as MRI are crucial in evaluating pelvic masses. MRI provides detailed soft tissue contrast, allowing for assessment of the mass's extent and relationship to adjacent structures [11]. In this patient, MRI revealed the mass's significant size and its displacement of the bladder and rectum without obvious invasion, aiding in surgical planning.

The management of pelvic masses in NF1 patients is challenging. Surgical resection is often the primary treatment, but complete removal may be difficult due to the size of the mass and proximity to vital structures [12,13]. In cases where the mass causes significant symptoms or complications like urinary retention, surgical intervention becomes necessary [12]. Our patient underwent total pelvic exenteration, which, while radical, was required due to the extensive involvement of the mass.

This case underscores the need for clinicians to maintain a high index of suspicion for unusual causes of urinary retention in young males. NF1 patients presenting with urinary symptoms should be evaluated for possible pelvic neurofibromas or other masses. Early detection and intervention can prevent complications and improve patient outcomes [13]. This case also highlights the significance of a multidisciplinary approach in managing complex cases involving pelvic masses and NF1. Collaboration among urologists, oncologists, radiologists, and surgeons is essential to provide comprehensive care [14].

## Conclusions

Acute urinary retention in young males should prompt consideration of uncommon aetiologies, such as pelvic masses, after the exclusion of common causes. Patients with NF1 are particularly at risk for developing tumours that may present atypically, highlighting the need for clinicians to maintain a high index of suspicion in this population. Thorough evaluation, including advanced imaging, is essential for early identification and management, potentially reducing complications. Given the complexities involved in diagnosing and managing NF1-related pelvic masses, multidisciplinary collaboration is vital to ensure comprehensive care.
